# Chronometry on Spike-LFP Responses Reveals the Functional Neural Circuitry of Early Auditory Cortex Underlying Sound Processing and Discrimination

**DOI:** 10.1523/ENEURO.0420-17.2018

**Published:** 2018-07-03

**Authors:** Arpan Banerjee, Yukiko Kikuchi, Mortimer Mishkin, Josef P. Rauschecker, Barry Horwitz

**Affiliations:** 1 Cognitive Brain Dynamics Lab, National Brain Research Centre, Gurgaon, Haryana 122052, India; 2 Brain Imaging and Modeling Section, National Institute on Deafness and Other Communication Disorders, National Institutes of Health, Bethesda MD 20892; 3 Institute of Neuroscience, Newcastle University Medical School, Newcastle upon Tyne, NE1 7RU, United Kingdom; 4 Laboratory of Neuropsychology, National Institute of Mental Health, Bethesda MD 20892; 5 Department of Neuroscience, Georgetown University Medical Center, Washington, DC 20057

**Keywords:** auditory cortex, belt, core, functional connectivity, latency, spike LFP

## Abstract

Animals and humans rapidly detect specific features of sounds, but the time courses of the underlying neural response for different stimulus categories is largely unknown. Furthermore, the intricate functional organization of auditory information processing pathways is poorly understood. Here, we computed neuronal response latencies from simultaneously recorded spike trains and local field potentials (LFPs) along the first two stages of cortical sound processing, primary auditory cortex (A1) and lateral belt (LB), of awake, behaving macaques. Two types of response latencies were measured for spike trains as well as LFPs: (1) onset latency, time-locked to onset of external auditory stimuli; and (2) selection latency, time taken from stimulus onset to a selective response to a specific stimulus category. Trial-by-trial LFP onset latencies predominantly reflecting synaptic input arrival typically preceded spike onset latencies, assumed to be representative of neuronal output indicating that both areas may receive input environmental signals and relay the information to the next stage. In A1, simple sounds, such as pure tones (PTs), yielded shorter spike onset latencies compared to complex sounds, such as monkey vocalizations (“Coos”). This trend was reversed in LB, indicating a hierarchical functional organization of auditory cortex in the macaque. LFP selection latencies in A1 were always shorter than those in LB for both PT and Coo reflecting the serial arrival of stimulus-specific information in these areas. Thus, chronometry on spike-LFP signals revealed some of the effective neural circuitry underlying complex sound discrimination.

## Significance Statement

Primary auditory cortex (A1) and lateral belt (LB) areas are key subdivisions of auditory cortex. A1 plays crucial role in processing of simple stimuli such as pure tones (PTs), whereas LB for processing of complex sounds. Both areas receive direct inputs from medial geniculate nucleus (MGN) and have recurrent connections. Nonetheless, the functional connectivity patterns between these subdivisions while processing different sound categories are poorly understood. Using simultaneous spike-LFP recordings, our study reveals that information about the presence of stimuli in the environment arrives concurrently in A1 and LB; however, the information related to neuronal discrimination may arrive at different times, indicating that both parallel and serial information transmission pathways exist and their presence is guided by the context of the task.

## Introduction

Simple auditory stimuli such as pure tones (PTs) are represented as tonotopic maps in primary auditory cortex (A1; Hind, 1953; Merzenich et al., 1976; Romani et al., 1982; Morel et al., 1993), whereas belt areas, lateral and medial to the core, while still showing cochleotopic organization, process more complex features of sounds (Muscari et al., 1990; Rauschecker et al., 1995; Tian and Rauschecker, 2004; Recanzone, 2008; Kuśmierek and Rauschecker, 2009; Niwa et al., 2013; Kikuchi et al., 2014). The core is primarily defined based on the thalamic connections from the ventral division of the medial geniculate nucleus (MGN) and reciprocally connected with the adjacent subdivisions of the belt (Hackett, 2011; Scott et al., 2015). Thus, the functional organization of complex sounds in core and belt can be hypothesized to follow a serial processing stream, from core to belt, somewhat analogous to V1 and the V2/V4/MT areas of the visual system (Tian et al., 2013). At the same time, direct inputs from the MGN to these brain areas point to parallel processing pathways (Rauschecker et al., 1997), which continue further downstream (Sheline et al., 2010). Finally, demands of a task, such as sound localization, categorization, and discrimination, can also govern the serial versus parallel characterization of processing (Ahveninen et al., 2006, 2013; Bizley and Cohen, 2013).

**Figure 1.**
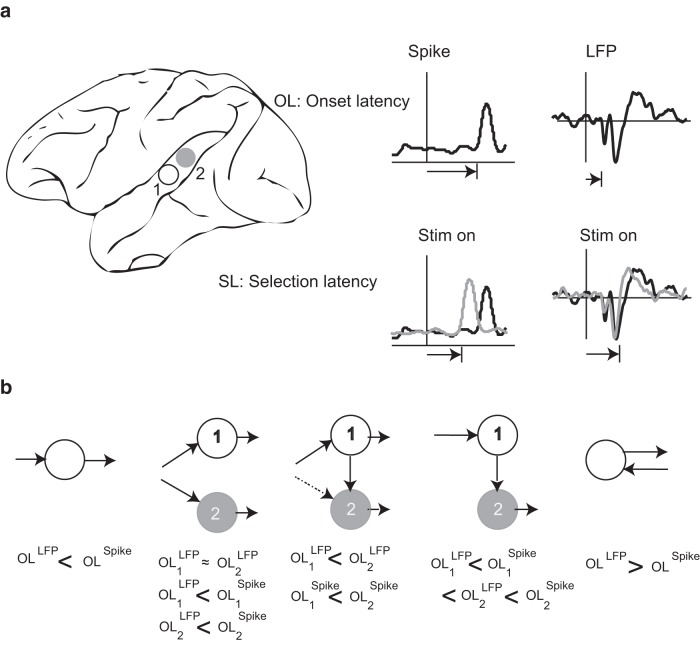
***A***, Simultaneous recordings from two arbitrary brain areas 1 and 2. On the right, we illustrate the definition of onset latencies (OLs) and selection latencies (SLs) by plotting the spike response (left panel) and LFP (right panel) from each recording site. OL is computed using an event as model 1 in the AccLLR framework (Banerjee et al., 2010) and prestimulus baseline as model 2 (see Eq. 1). SL is computed using PT stimulus as model 1 and Coo as model 2. ***B***, The effective network architectures inferred from different OL values. The solid lines reflect the effective network connections, whereas the dotted lines indicate a less likely connection that can be inferred from latency measures.

Chronometry of input and output related processing events in candidate brain areas is a useful technique for functional network identification (Kreiman et al., 2006; Nielsen et al., 2006; Monosov et al., 2008; Banerjee et al., 2010, 2012). While neuronal spike discharge is used as a measure of output processing in a putative brain area (Krüger and Becker, 1991; Middlebrooks et al., 1994; Nawrot et al., 2003; Buzsáki et al., 2012), local field potentials (LFPs) may carry information about the inputs coupled with local neuronal processing that need not be input specific, in a particular brain area (Gusnard et al., 2001; Nielsen et al., 2006; Buzsáki et al., 2012) and by extending this principle to multiple brain areas, aspects of the functional circuitry underlying behavior can be revealed (Hung et al., 2005; Banerjee et al., 2012; Fig. 1). Conceptually, shorter latencies in one area compared to another reflect faster processing and greater relevance of the former brain area and thus indicate more efficient neuronal coding (Gawne et al., 1996; Van Rullen and Thorpe, 2001; Bendor and Wang, 2008). Additionally, the timing of input versus output of information processing in an area can be used to infer the role of this area in processing of a particular type of signal as well as the functional pathways involved in processing of the signal (DiCarlo and Maunsell, 2005).

Spike trains and LFPs in auditory core exhibit comparable frequency tuning (Kayser et al., 2007). On the other hand, there is evidence suggesting that the cochleotopic organization of belt areas is less precise, as observed in spike-LFP responses (Guo et al., 2012). Hence, identifying the temporal markers of inputs and outputs involved in information processing in auditory core and belt across single units and populations can help reveal the functional specificity of the respective areas. Extending this line of reasoning, Camalier and colleagues computed neuronal onset latencies at different locations along the auditory cortical pathways and reported that dorsal stream locations have shorter latencies, whereas the ventral locations exhibit increasingly longer latencies as one proceeds from lower to higher-order processing (Camalier et al., 2012). This result conforms with human studies using magnetoencephalography and transcranial magnetic stimulation (Ahveninen et al., 2006, 2013) as well as with other monkey studies (Scott et al., 2011; Kuśmierek and Rauschecker, 2014). Kikuchi et al. (2014) reported that PT-related spike onset latencies were longer in lateral belt (LB) than in auditory core, which is consistent with the notion that auditory core is at a lower hierarchical level within cortex than LB. However, do the two areas receive information about stimulus presence concurrently? Furthermore, are the finer features that allow discrimination of one signal from another represented in the neural codes hierarchically?

To address these questions, we recorded spike and LFP responses simultaneously from A1 and LB of two adult macaques while they performed an auditory Go/No-go discrimination task. We computed trial-by-trial onset Latency, time locked to stimulus onset, and selection latency, the earliest time at which neural responses between PTs and Coos significantly differ. Computing these measures in different subdivisions of auditory cortex, we could tease out the functional network mechanisms involved in sound processing and discrimination.

## Materials and Methods

### Animal preparations and behavioral task

Two adult male rhesus macaques (*Macaca mulatta*, weighing 7.5–11.5 kg) participated in this study. Animal care and all procedures were conducted in accordance with the National Institutes of Health guidelines and were approved by the Georgetown University Animal Care and Use Committee. Animals were prepared for chronic awake electrophysiological recordings under aseptic conditions. Each animal was anesthetized and a head post and recording chamber were attached to the dorsal surface of the skull with a guidance of MRI obtained with a 3T scanner (0.5 mm voxel size, Siemens Tim Trio). The recording sites in this study cover the auditory core (A1) and the auditory LB region [the middle lateral (ML) and anterolateral (AL)]. We followed identical methods for assigning the recording sites to either A1 or LB as described in Kikuchi et al. (2014).

Electrophysiological experiments were conducted in a single-walled acoustic chamber (Industrial Acoustics Company) installed with foam isolation elements (AAP3, Acoustical Solutions). The animal sat in a monkey chair with its head fixed, facing a speaker located one meter directly in front of it in a darkened room. The animal was trained to perform an auditory discrimination task, in which a single positive stimulus (S+), consisting of a 300-ms pink-noise burst (PNB), was pseudo-randomly interspersed among negative stimuli (S-), consisting of all other stimuli, for 20% of the trials. The animal initiated a trial by holding a lever for 500 ms, triggering the presentation of one of the acoustic stimuli, was required to release the lever within a 500-ms response window after the offset of the S+ to get a water reward (∼0.2 ml) that followed by a 500-ms intertrial interval (ITI). Lever release in response to S- prolonged the 500-ms ITI by 1 s (timeout). The average interstimulus interval was 2.3 ± 0.45 s (mean ± SD). The detailed procedures for the animal preparations, behavioral task, and data collection were the same as those described in Kikuchi et al. (2014).

### Sound preparation and stimuli

The sound wave form signals were sent from the CORTEX dual-computer system through a 12-bit D/A converter (CIO-DAS1602/12, ComputerBoards), and then amplified, attenuated, and delivered through a free-field loudspeaker (Reveal 6, Tannoy) with a flat (±3 dB) frequency response from 63 Hz to 51 kHz.

The monkey vocalizations (“Coo” calls) were recorded in Morgan Island using a directional microphone (ME66 with K6 powering module, Sennheiser, frequency response at 40–20,000 Hz ± 2.5 dB) with a solid-state portable recorder (PMD670, Marantz Professional) at a sampling rate of 48 kHz (Laboratory of Neuropsychology, National Institute of Mental Health). PTs and PNBs were created using Adobe Audition 1.5 at a sampling rate of 48 kHz (32 bit). All stimuli had a 300-ms fixed duration, including the monkey vocalizations, gated with a 5-ms rise/fall linear ramp. The stimuli were normalized across all stimuli by recording the stimuli played through the stimulus presentation system and filtering the recorded signal on the basis of Japanese macaque audiograms (Jackson et al., 1999), and using the maximum root mean square (RMS) amplitude during a sliding window of 200-ms duration and presented at ∼70-dB SPL. Details of the sound equalization method were described by Kuśmierek and Rauschecker (2009).

**Figure 2.**
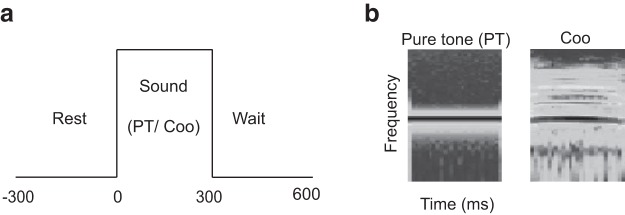
Experimental design, Go/No-go task. ***A***, Monkey waits during a rest period with hands on a lever and attends to the stimuli (PT, Coo, or pink noise; presentation time, 300 ms). To obtain a water reward, the monkey must release the lever when pink noise is presented. The next trial starts 600 ms after the previous stimulus onset. ***B***, The spectrogram (time, frequency, and power) of PT and Coo stimuli. The frequency of PTs matches the fundamental frequency of the Coo.

The positive stimulus was a pink noise, a response to which led to a reward, whereas the negative stimuli were made up of both PTs and Coo vocalizations. A stimulus set comprised of 10 PTs and 10 pitch-matched Coos, in which the fundamental frequency (F0) of the Coo was match to the corresponding frequency of PT using the pitch-shift function in Adobe Audition 1.5 (Fig. 2). The frequency of PTs and the F0 of the Coos ranged from G3 (196 Hz) to C#8 (4435 Hz) in six semitone steps. In each recording session, the stimuli were presented in pseudorandom order with at least 15 trials per stimulus.

### Data collection and preprocessing

**Figure 3.**
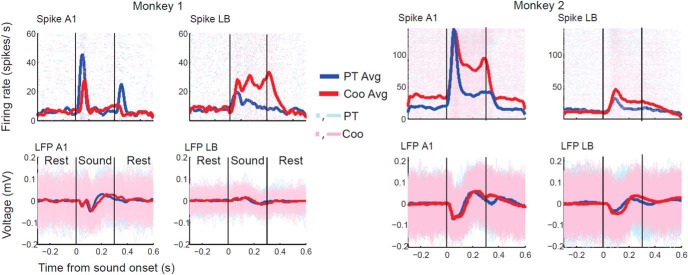
One representative session from each monkey, where simultaneous recordings from two areas spikes and LFP could be obtained. First row indicates spike rasters (cyan and magenta dots) and firing rates (blue and red) computed using Gaussian smoothing (10-ms window) for PT (cyan/blue) and Coo (magenta/red) stimuli. The second row depicts the trial-by-trial LFP waveforms using the same color code as for spikes. The averaged LFP responses are plotted in blue and red. The spike-LFP responses in two auditory cortical areas A1 and LB were recorded during the same session in each monkey.

Multiple guide tubes carrying up to 4 tungsten microelectrodes (0.5–3.0 MΩ, epoxylite insulation, FHC) was lowered into the target cortical sites identified on the MRI scans. Each electrode was independently advanced using a remote-controlled hydraulic, four-channel customized multidrive system (NAN-SYS-4, Plexon. Inc.). For the spike trains, raw signals were filtered with a bandpass of 150–8000 Hz, further amplified, and then digitized at 40 kHz. For the LFP, the raw voltage traces were filtered between 0.7 and 500 Hz, amplified, and digitized at 1 kHz. For further analyses, the LFP data were low-pass filtered at 100 Hz. Time stamps for stimulus presentation timings, behavioral response, and reward delivery were sent through DOS-CORTEX dual computer system (CIO-DAS1602/12, CIO-DIO_2_4, ComputerBoards). Spikes were sorted by real-time acquisition programs using template matching and Principal Component Analysis (PCA) methods (RASPUTIN, Plexon). We focused on trials in which simultaneous spike-LFP recordings were obtained from both monkeys in both core and LB areas. Overall, we accumulated data from 29 sessions in Monkey1 and 27 sessions from Monkey2, for a total of 56 sessions, where a session was defined as a group of trials for which we obtained simultaneous spike train recordings from one neuron in A1 and one in LB. Two sessions may have different single cells (spike-sorted) but the same LFP representation. We aggregated all 23 fundamental frequencies presented to the monkeys under a single category called PT trials. Similarly, all F0-matched monkey calls were categorized as Coos. This enhanced the statistical power of our analytical framework but did not adversely affect the main goals of the study. Hence, to increase the statistical power of our analysis, we chose to categorize all PT trials as one block and the F0-matched Coo trials as a different block. Firing rates reported in Figure 3 were computed from binary spike rasters by applying Gaussian smoothing with a 10-ms window on the averaged peristimulus histogram (PSTH) with a bin size of 1 ms. The mean evoked LFP wave form was calculated by averaging LFPs across trials.

### Trial-averaged latency analysis

**Figure 4.**
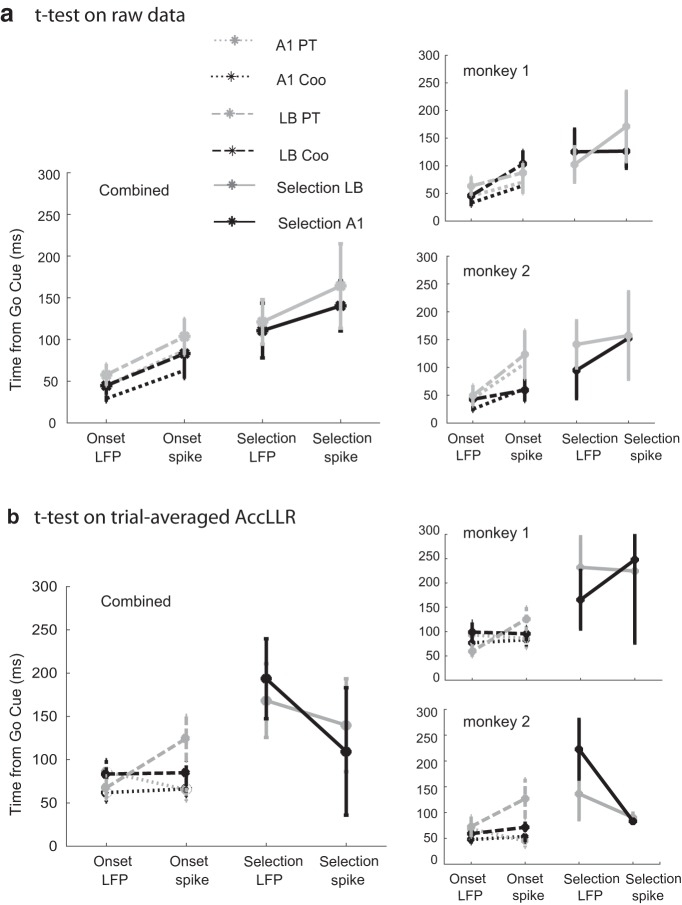
***A***, Estimation of trial-averaged onset and selection latency using ms-by-ms *t* test on raw time series. For spikes, the binary time series was transformed to a SDF by convolving single trial spike trains with assymetric exponential functions having different growth and decay time constants, 1 and 20 ms, respectively, following Thompson et al. (1996). A ms-by-ms *t* test was performed on the distribution SDFs in a given session from different conditions (see text for details). For onset, prestimulus rest period was used to compute the SDF. Analyses were performed across both monkeys and for each monkey individually. Error bars were plotted at 95% significance levels. ***B***, Estimation of trial-averaged onset and selection latency from AccLLR distributions using ms-by-ms *t* test; *p* = 0.01 was chosen as threshold for significance.

Histograms of binary spiking events were computed using 1-ms bins and were convolved with growth-decay functions (Thompson et al., 1996; Monosov et al., 2008) to compute continuous spike density functions (SDFs). Time constants for growth phase, $\tau_{g}=1ms$ and for decay phase, $\tau_{d}=20ms$ were used to compute the spike density fuctions following Thompson et al. (1996). A ms-by-ms *t* test was applied to the two SDFs either within different temporal segments of the same trial (for onset) or between trials from different conditions (for discrimination) to obtain the onset and selection latencies, respectively, over an entire session (Fig. 4). As LFPs are continuous signals, the raw LFP traces (band-passed between 0 and 200 Hz) were used to compute onset and selection latencies. Pairwise Wilcoxon rank-sum tests were performed to establish significant effects. We report the statistical analysis performed on data pooled from both monkeys and set a threshold of *p* = 0.01 for estimating significance. We set the threshold to this slightly conservative value since there were a large number of trials in each session that were available for the trial-by-trial analysis (see below).

### Trial-by-trial AccLLR analysis

**Figure 5.**
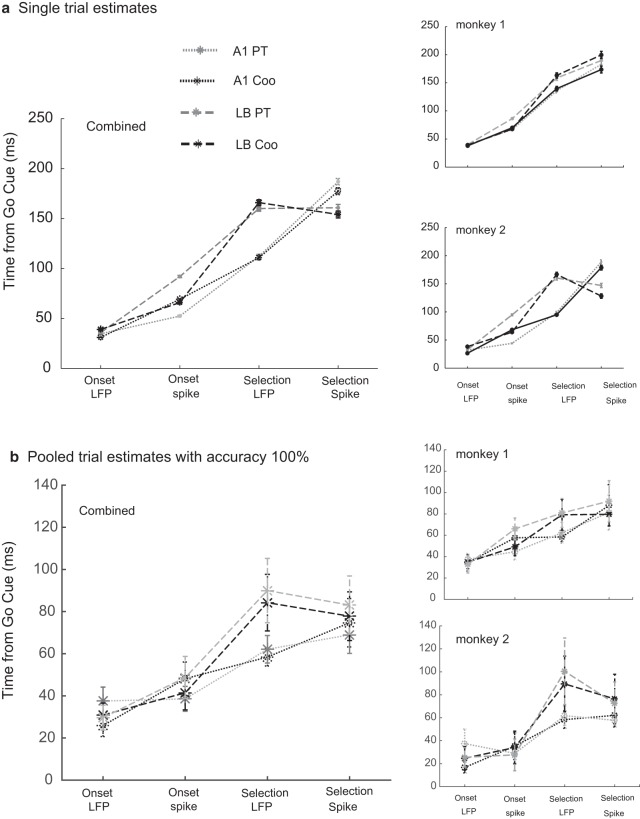
***A***, Estimation of trial-by-trial onset and selection latency using AccLLR. We follow the same pattern of presentation as in Figure 4 and report the group-level analysis and individual monkey analysis. Error bars were computed at 95% significance levels by pooling all trials and sessions in a monkey. See text for details of Methods. ***B***, Estimation of trial-by-trial onset and selection latency using AccLLR on pooling all trial information within a stimulus category to a single trial in each session and setting accuracy to 100%. Error bars were computed at 95% significance levels.

Spike trains and LFPs follow different statistical properties and hence the estimation of single-trial latencies from these two signals requires a unified framework (Banerjee et al., 2010, 2012). AccLLR addresses this issue and computes spike-LFP latencies trial-by-trial (Fig. 5). AccLLR is a model-based framework that requires two competing models of observations. We have used time-varying firing rate models for spiking (inhomogeneous Poisson process) and time-varying continuous means and standard deviations (Gaussian process) for LFP signals. For further discussion on different kinds of models, see Banerjee et al. (2010, 2012). Once the model parameters (time-dependent firing rate for spikes and mean and standard deviation for LFP) are computed from a set of training trials, the likelihood that the time series for a test trial (binary spike trains for spikes, continuous wave form for LFP) belongs to model 1 or model 2 can be computed. Finally, raw spike trains and continuous LFPs can be transformed into the space of accumulated log-likelihood ratios by first calculating likelihood ratios (LRs)

(1) $$LR\left(t\right)=\frac{P\left(x\left(t\right)\right|Model1)}{P\left(x\left(t\right)\right|Model2)}$$

where x(t) is the data point at which LR is computed. To compute the *LRs*, we use the leave-one-out principle. The trial at which LR was computed does not contribute to obtaining the model parameters. The rest of the trials are used in model development. This was done to minimize the bias of any particular model.

**Figure 6.**
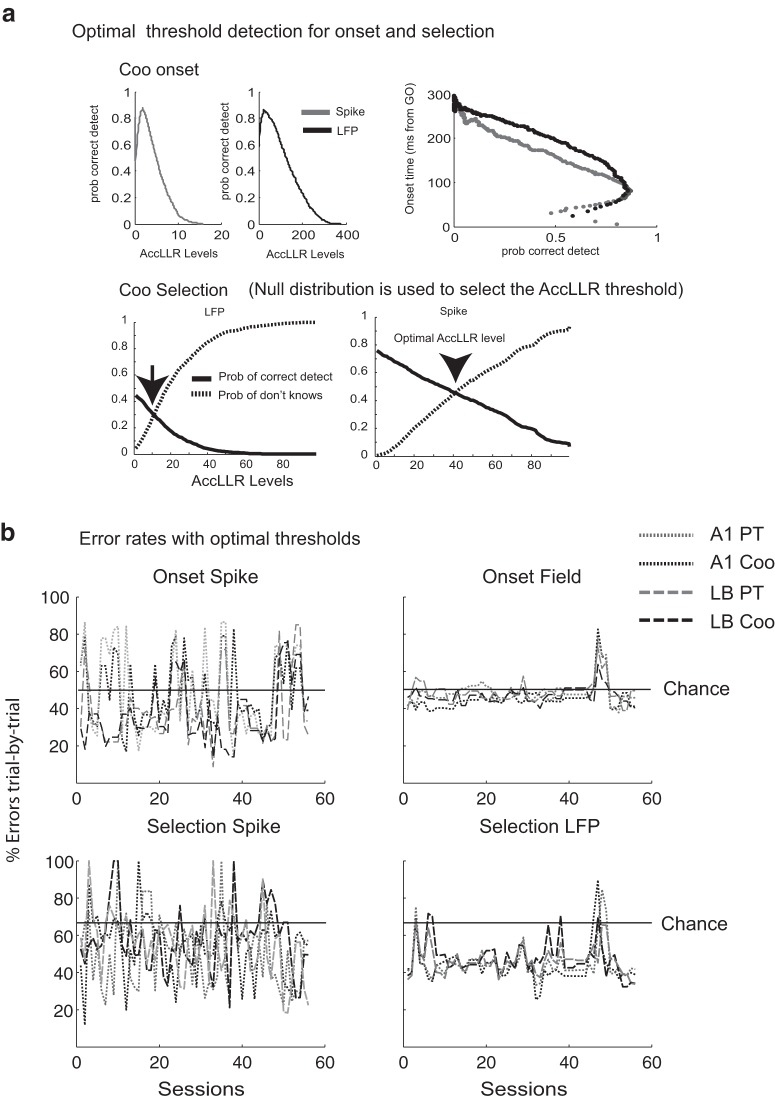
Decoding performance using AccLLR. ***A***, Setting up the bounds of accumulation is an integral part of AccLLR analysis. The probability of correct detection varies with where the bound is set for both spikes and LFPs. Furthermore, the onset latency also varies with the selection of thresholds and, consequently, with the probability of correct detection. Optimal onset latency detection is defined when the threshold for the false positive rate for prestimulus data (null) equals or exceeds the detection of true positives from event (poststimulus period) data. For selection latency, there are three possibilities: PT, Coo, or don’t know (baseline). Here, the optimal threshold was chosen when the probability of correct detection matched the probability of don’t knows from the rest period (null) data. ***B***, Error rates of decoding from spikes (1st column) and LFPs (2nd column). Error rates for onset (1st row) and selection latency (2nd row) are also shown in matching color codes. Note that *y*-axis is error, lower error indicates better performance.

By integrating the natural logarithm of $LR\left(t\right)$over time we obtain accumulated log-likelihood ratios$\left(AccLLR\left(t\right)\right)$, which follow a drift-diffusion process (Gold and Shadlen, 2001; Eckhoff et al., 2008; Banerjee et al., 2010). Thus, the difference in statistical properties of spike trains and LFPs become inconsequential in the space of AccLLRs, which unifies these measurements. Latencies are computed setting bounds specific to a model (1 or 2) of AccLLRs (Fig. 6).

An important aspect of the AccLLR framework is that it sets the bounds on the accumulation of integrated log-likelihood ratios, ordinarily done using the sequential probability ratio test (SPRT; Wald and Wolfowitz, 1947). Under this framework, accumulated log-likelihood ratios obtained using Equation 1 reaches a decision threshold after “sufficient” information has been collected. Alternatively, information is sufficient to make a decision when a certain threshold is reached. At an asymptotic limit, a mathematical relationship connecting the location of bounds of AccLLR accumulation to false positive and false negative rates can be expressed (Wald and Wolfowitz, 1947).

For the purpose of decoding latencies within a biologically relevant time, we chose a data-driven approach to set the bounds on AccLLR accumulation (Banerjee et al., 2010). For a given poststimulus event as model 1, there are two possibilities for detection within a finite time, viz, whether the event is correctly detected (true positive) or no detection is possible (false negative). On the other hand, for the prestimulus baseline (null) as model 2, either correct (true negatives) or incorrect (false positives) assignment is made. For setting a bound for onset detection, we chose an optimum threshold for which the false positive rate for null data equals or exceeds the detection of the true positive rate on event data. For setting bounds for selection latency detection, we first computed the AccLLRs for a “null” period (prestimulus baseline), 300 ms from the start of a trial. There are three potential outcomes: AccLLR reaches (1) an upper threshold corresponding to hit rate for model 1; (2) a lower threshold corresponding to hit rate for model 2; and (3) does not reach either threshold (“don’t know”). Again, the threshold for detecting model 1 was chosen at an optimal point where the probability of don’t knows exceeds the hit rate for model 1. Similarly, the threshold for detecting model 2 was chosen at an optimal point where the probability of don’t knows exceeds the hit rate for model 2 (Fig. 6). For further details, see Banerjee et al. (2012).

While decoding latencies at the level of single trial brings us close to revealing the true nature of neural processing occurring at a realistic time scale, nonetheless, the process of choosing a threshold is impacted by speed-accuracy trade off, meaning a lower threshold can make detection faster while increasing the false positives, and on the other hand a higher threshold can increase accuracy but also increase the onset and selection latencies. Hence, to check that the consistency of latency results are extended to situations where accuracy is set at 100%, we pooled all log-likelihood ratios from all trials within a session to create a pseudo-trial. Accumulated log-likelihood ratios were computed on this trial for each detection context, onset and selection. AccLLR threshold for onset detection was chosen to be the maximum AccLLR reached by a null trial. Similarly, AccLLR threshold for selection of one category of stimulus (model 1) was determined by the maximum reached by AccLLR from the second category (model 2).

## Results

Spike-LFP recordings were obtained simultaneously from two brain areas, A1 in the auditory core and LB. Our recordings in LB came from two subdivisions, AL and ML belt areas. Here, we were interested in trials where simultaneous spike-LFP recordings were obtained from both monkeys in both A1 and the LB areas. We accumulated 29 sessions in Monkey1 and 27 sessions in Monkey2, totaling 56 sessions in which simultaneous spike-LFP recordings were obtained in A1 and LB. We computed the onset latency of the neural response of either spike or LFP using the method of accumulated log-likelihood ratios (AccLLR; for details, see Banerjee et al., 2010). According to this framework, for single/multiunit spiking activity, the baseline can be the background firing rate during the prestimulus period. Analogously, in the case of LFP, the baseline can be the distribution of voltage traces during the prestimulus period. We computed the timing of information processing events from trial-by-trial spike-LFP data (for further details of the method, see Banerjee et al., 2010, 2012).

Monkeys performed the auditory discrimination task in a Go/No-go setup illustrated in Figure 2. Monkeys were trained to discriminate different kinds of sounds (all negative, or No-go cues) from a pink noise stimulus (positive, or Go cue), which, when responded to, resulted in a water reward. Onset latency and selection latencies were computed from spike-LFP responses. Onset latency characterized the boundaries of a processing stage required for encoding the presence of sound in the environment, thereby a measure of stimulus-related processing. On the other hand, selection latency characterized the boundaries of a processing stage involved in coding the presence of a specific sound in the environment, hence yielding a measure of stimulus-specific processing. Figure 3 illustrates an example recording session in each monkey. In Monkey1, we observed a transient increase in spike frequency around the beginning and end of the stimulus in A1, whereas we saw sustained spiking responses in LB. Simultaneously, a difference in LFP waveforms is observed during stimulus presentation for the two stimulus categories. In Monkey2, we observed sustained firing in A1 following a transient rise of spike rate at stimulus onset. Furthermore, LFP differences were observed primarily between two stimulus categories in a period following the termination of stimulus presentation. These examples illustrate the diversity and complexity of spike/LFP responses across different recording sessions in both A1 and LB.

### Chronometry on spike-LFP responses

We computed neuronal response latencies for onset and discrimination using two approaches: a traditional trial-averaged approach and single-trial AccLLR analysis (Banerjee et al., 2010) of spike-LFP data. The former gives a broad summary of the results, and the latter helps in addressing the between-trial variability in neural signals and gives a more consistent account of neuronal information processing. In the first approach, a ms-by-ms *t* test (Monosov et al., 2008) was used to compute trial-averaged measures of latencies. This approach is a standard one, used by most investigators. In the second approach, the AccLLR framework was used to compute trial-by-trial latencies of onset and selection (Banerjee et al., 2012). Additionally, we applied ms-by-ms *t* test on trial-by-trial AccLLR distributions for each session to compute the trial averaged latencies. In both cases, simultaneously collected data from two brain regions (A1 and LB) were used. We report statistics performed over all sessions from two monkeys in both the text (*p* values) and Table 1 (mean and SEM).

**Table 1.** Mean neuronal latencies for onset and selection of auditory stimulus with SEM reported in parentheses

Signal	Area	Onset (ms)		Selection (ms)		
		PT (56)	Coo (56)	PT-Coo (56)		
**LFP**	**A1**	**45 (4.0)**	**29 (1.93)**	**111 (16.86)**		**Trial averaged**
	*LB*	*58 (6.76)*	*45 (5.65)*	*121 (13.77)*		**(*t* test on raw data)**
**Spike**	**A1**	**87 (10.36)**	**63 (5.47)**	**140 (15.55)**		
** **	*LB*	*103 (13.11)*	*83 (8.86)*	*164 (26.15)*		
** **	** **	**PT** (56)****	**Coo** (56)****	**PT-Coo** (56)****		
**LFP**	**A1**	**60 (7.13)**	**51 (5.8)**	**155 (22.57)**		**Trial averaged**
	*LB*	*57 (5.59)*	*55 (7.37)*	*170 (22.19)*		**(*t* test on AccLLR)**
**Spike**	**A1**	**67 (10.54)**	**74 (6.15)**	**89 (16.54)**		
** **	*LB*	*87 (11.76)*	*94 (11.43)*	*106 (20.91)*		
** **	** **	**PT **(15319)****	**Coo **(15326)****	**PT **(13825)****	**Coo **(14035)****	
**LFP**	**A1**	**35 (0.22)**	**31 (0.21)**	**113 (1.04)**	**111 (1.03)**	**AccLLR: trial-by-trial**
	*LB*	*36 (0.32)*	*39 (0.29)*	*161 (1.38)*	*167 (1.48)*	
**Spike**	**A1**	**52 (0.40)**	**69 (0.46)**	**187 (1.64)**	**178 (1.77)**	
** **	*LB*	*92 (0.56)*	*66 (0.33)*	*163 (1.67)*	*155 (1.77)*	
****LFP****	**A1**	**38 (3.4)**	**26 (2.7)**	**62 (3.1)**	**58 (2.32)**	**AccLLR: 100% accuracy matched pooled trials**
	***LB***	*30 (3.06)*	*31 (2.9)*	*90 (8.0)*	*84 (7.15)*		
****Spike****	**A1**	**39 (3.4)**	**48 (4.45)**	**69 (5.48)**	**75 (7.5)**	
	***LB***	*49 (5.3)*	*41 (4.3)*	*83 (8.02)*	*78 (7.33)*	

The sample sizes are indicated at the beginning of each column in parentheses and underlined. The numbers for A1 are presented in bold and LB in italics for ease of view.

#### Trial-averaged latencies from raw data

Ms-by-ms *t* test was applied to raw LFP traces and spike distribution functions (for details, see Materials and Methods) to extract spike-LFP latencies as followed by an earlier study (Monosov et al., 2008). Analyses of the combined data from both monkeys are presented in Figure 4*A*, Table 1 (results from each monkey are also presented separately in Fig. 4) for a sample size of 56 sessions (29 for Monkey1 and 27 for Monkey2). Note that we rounded latencies to whole numbers for reporting group averages and that *p* = 0.01 was chosen as the threshold in pairwise *t* tests used to evaluate the statistical significance of both trial-averaged and AccLLR analysis across stimuli categories and brain areas. For PTs, mean LFP onset latency in A1 (45 ms) was not significantly different (*p* = 0.22) from mean LFP onset latency in LB (57 ms). The same was true not true for Coo (*p* = 0.001, mean 29 ms in A1, 45 ms in LB). However, the stimulus-specific differences between LFP onset latencies (i.e., between PT and Coo) were significant in A1 (*p* < 0.01) but not in LB (*p* = 0.02).

Mean spike onset latencies for PT were 87 ms in A1 and 103 ms in LB. Mean spike onset latencies for Coo were 63 ms in A1 and 83 ms in LB. No stimulus-specific differences were observed for spike onset latencies (PT vs Coo) in either A1 (*p* = 0.1) or LB (*p* = 0.25). However, spike-LFP onset latency differences in A1 and LB were significant for both stimuli (PT and Coo; *p* < 0.01 for all four comparisons). These results suggest that inputs arrive at A1 and LB from a shared source and that there is considerable parallel processing across the two areas.

Trial-averaged selection latencies are computed from ensembles of trials belonging to two stimulus categories. That is, one selection latency value is defined for two stimulus categories for each session of recording. LFP selection latencies in A1 and LB were not significantly different (*p* = 0.44, mean 111 ms in A1 and 121 ms in LB; detailed statistics are presented in Table 1). Similarly, spike selection latencies across A1 and LB were also not significantly different (*p* = 0.41, mean 140 ms in A1 and 164 ms in LB).

Spike-LFP selection latency differences were not significant in LB (*p* < 0.01) and A1 (*p* = 0.03) with the threshold level set at *p* = 0.01. Thus, hierarchical stimulus processing from A1 to LB cannot be inferred from this analysis. A largely similar pattern of results was observed when the analysis was repeated for each monkey separately (Fig. 4*A*).

#### Trial-averaged latencies from AccLLR estimates

AccLLR at the level of single trials is a probabilistic method dependent on accumulation evidences. Inherently, it has a “slowness” incorporated to it which is further dependent on signal-to-noise ratios. Hence to get a sense of the speed-accuracy trade-off that affects AccLLR analysis we applied ms-by-ms *t* tests on distribution of AccLLRs to compute the trial-averaged latencies (Fig. 4*B*; Table 1). This will give an estimate of latencies that can be achieved with maximum accuracy using AccLLR analysis for the ensemble of trials and sessions.

Mean LFP onset latency for PT was 60 ms in A1 and 57 ms in LB and was not significantly different (*p* = 0.61). Mean LFP onset latency for Coo was 51 ms in A1 and 55 ms in LB and was not significantly different (*p* = 0.09). The stimulus-specific differences between LFP onset latencies (i.e., between PT and Coo) were not significant in A1 (*p* = 0.06) and LB (*p* = 0.02).

Mean spike onset latencies for PT was 67 ms in A1 and 87 ms in LB, which were not statistically significant (*p* = 0.73). Mean spike onset latencies for Coo were 74 ms in A1 and 94 ms in LB, which were not significantly different (*p* = 0.62). We did not find any stimulus-specific differences for spike onset latencies (PT vs Coo) in A1 (*p* = 0.45) and LB (*p* = 0.47). Spike-LFP latencies were significant different for Coo in A1 (*p* = 0.01) but not for PT (*p* = 0.58). Spike-LFP latencies were not significantly different in LB for both Coo (*p* = 0.03) and PT (*p* = 0.02) stimuli.

LFP selection latencies in A1 (155 ms) and LB (170 ms) were not significantly different (*p* = 0.25). Analogously, spike selection latencies in A1 (89 ms) and LB (106 ms) were not significantly different (*p* = 0.24). No significant differences were found between spike-LFP selection latencies in A1 (*p* = 0.06) and LB (*p* = 0.25).

#### Trial-by-trial AccLLR analysis

AccLLR (Banerjee et al., 2010) was used to compute trial-by-trial onset and selection latencies (Fig. 5; Table 1). Spike and LFP data were transformed to AccLLR space using inhomogeneous Poisson models for spikes and Gaussian models for LFP. Latencies were computed by setting decision bounds on AccLLR time series. For onset latency estimation, model 1 was applied to spike/LFP data during the stimulation period and model 2 to prestimulus baseline; for selection latency, model 1 was applied to PT trials and model 2 to Coo trials (for details, see Materials and Methods). Latencies were estimated using the leave-one-out rule, where model parameters were estimated from all other trials leaving aside the one for which the latency was being computed. Two major advantages of using the AccLLR method were that we could reduce the variability observed in the trial-averaged analysis and that the stimulus-specific selection latencies could be computed trial-by-trial. On the other hand, a definition of single selection latency encompasses at least two trial categories for trial-averaged analysis. The AccLLR analysis had orders of magnitude higher sample sizes than those in the trial-averaged analysis (Table 1). Theoretically, unlike the raw data, AccLLRs from both spike and LFP follow the same statistical distribution (for details, see Materials and Methods), hence spike-LFP comparisons are quantitatively valid. The mean and SEM for onset and selection latencies are reported in Table 1.

The mean LFP onset latencies in A1 and LB for PT stimuli were nearly identical (35 ms in A1, 36 ms in LB, *p* = 0.10). On the other hand, the mean LFP onset latency for Coos differed significantly in the two areas (31 ms in A1, 39 ms in LB, *p* < 0.01). Spike onset latencies differed significantly between A1 and LB for PTs (52 ms in A1, 92 ms in LB, *p* < 0.01). For Coos, the difference in spike onset latencies between A1 and LB is small but significant (69 ms in A1, 66 ms in LB, *p* < 0.01). Together, these results suggest that processing relatively simpler stimuli like PT can be supported by A1, whereas more complex stimuli such as Coo require resources of a higher order area such as LB. LFP onset latencies always preceded spike onset latencies in each area and each stimulus category (*p* < 0.0001).

Interestingly, for either type of stimulus, LFP selection latencies were always shorter in A1 than in LB (for PT, means of 113 ms in A1 vs 161 ms in LB, *p* < 0.01; for Coo, 111 ms in A1, 167 ms in LB, *p* < 0.01), whereas spike selection latencies were always shorter in LB than in A1. For PT, spike selection latency was 187 ms in A1 and 163 ms in LB, *p* < 0.01; and for Coo, 178 ms in A1 vs 155 ms in LB, *p* < 0.01. Most interestingly, for Coo the LFP selection latency (167 ms) lagged the spike selection latency (155 ms) significantly (*p* < 0.01).

#### Estimates from pooled trials with 100% accuracy

To evaluate whether the pattern of results holds in a scenario where detection accuracy is 100% (thus taking into consideration the effects of speed-accuracy trade-off), we pooled all trials in a session to create a single trial in the log-likelihood space. Details of procedures of how thresholds were selected are described in Materials and Methods.

The LFP mean onset latencies for PT was very similar in A1 and LB (Table 1), a difference of 8 ms, which was not significant (*p* = 0.03). A similar pattern followed for Coo (*p* = 0.26). A1 seems to have lower LFP onset latency for PT (26 ms) compared to Coo (38 ms), but the effect was weak (*p* = 0.01). In LB, the LFP mean onset latencies were identical for PT (30 ms) and Coo (31 ms; *p* = 0.87). A similar pattern followed for mean spike onset latencies, and as well was observed for LFP. When spike-LFP latencies were compared except in A1 for PT where spike-LFP latencies were not different (*p* = 0.12), LFP latencies typically precede spike latencies.

Mean selection latencies for LFP were much lower than that obtained with single trial measures however the main pattern of LFP selection latencies being lower in A1 compared to LB was consistent (*p* < 0.0001). The mean spike onset latencies were in close proximity and none of the comparisons was significant at *p* = 0.01. Even the spike-LFP latency differences were not significant for individual selection contexts, for PT in A1 (*p* = 0.33), Coo in A1 (*p* = 0.1), PT in LB (0.60), and Coo in LB (*p* = 0.93).

#### Decoding performance

An important requirement in any decoding analysis framework is to control for the false positives and false negatives while setting thresholds for category distinction. In principle, the AccLLR test is optimal (Wald and Wolfowitz, 1947). Under conditions in which sufficient information is available or after infinite accumulation, the number of times any threshold is crossed is circumscribed by type 1 and type 2 errors. However, we are interested in latencies which would be biophysically relevant and computed using comparable statistical constraints on spike trains and LFP data. Detection of latencies within a finite time is constrained by a trade-off between accuracy and early detection (Fig. 6*A*). Hence, we have chosen a data-driven approach to set the optimal thresholds for AccLLR accumulation, details of which are provided in Materials and Methods (Fig. 6). Trial-by-trial onset and selection latency decoding performance were significantly worse than the chance level in most sessions (Fig. 6*B*). Error rates for most LFP sessions were below the chance level (probability of target detection is achieved by random selection) for both onset and selection. For the onset latency, there are only two detection scenarios, whether the signal can be classified as category 1 (the pattern of spike/LFP response to a stimulus) or category 2 (the animal is alert but not hearing any sound). Hence, the probability of detection by chance is 0.5. For selection latency, the probability of detection by chance is 0.67 since there are three possibilities in a given datum (PT, Coo, or prestimulus baseline). Figure 6*B* unambiguously illustrates that error rates for selection latency detection from spikes and LFPs were mostly lower than chance level indicating superior performance of the AccLLR technique. Typically, recording sites with good onset detection also yielded superior selection detection and decoding from LFPs were more reliable with more consistent error rates over sessions.

## Discussion

Using two measures, onset latency for detecting the presence of sound in the environment and selection latency for identifying stimulus-specific neural codes in primate auditory cortical areas we aim to characterize the functional pathways of underlying information processing. We observed a trend in which LFP onset/selection latencies were shorter than spike onset/selection latencies by applying ms-by-ms *t* test on time series data. However, the trial-averaged techniques do not allow the measure of stimulus-specific selection latencies since a distribution of PT trials is used to identify the time of selection from a distribution of Coo trials.

AccLLR analysis of our data refined the statistical significance of the trends and helped to mathematically define stimulus-specific selection latencies. In a trial-averaged analysis using the *t* test on raw data as well as AccLLRs, a single numerical value of selection latency was obtained for all trials within a session and by construction across two stimulus categories. Hence, not surprisingly, latencies computed by AccLLR exhibited variability that were orders of magnitude smaller than trial-averaged tests. Both trial-averaged analysis and AccLLR at the level of single trials as well as accuracy matched pooled trials yielded similar values for LFP onset latencies across A1 and LB. This reinforces the view that areas A1 and LB may process simple stimuli in parallel. Except in case of A1 and PT stimulus, all three onset scenarios had LFP latencies preceding spike latency when accuracy was matched. Proximity of spike and LFP latency typically indicates a central role of the recorded brain area in neuronal processing. Thus, our observations highlight area A1’s dominant role in coding PTs, whereas coding of complex stimulus such as Coo and in areas higher order than A1 are more mixed in nature. Selection latencies for each trial category can be only obtained from AccLLR analysis. Shorter LFP selection latencies for A1 than LB suggest information arrival in auditory brain areas can occur hierarchically. Interestingly both single-trial decoding as well as performance matched pooled trial analysis showed non-significant differences between LFP and Spike selection latency in LB; in particular the performance matched analysis revealed that LFP selection latencies had a trend of preceding spike selection latency thus reflecting a greater involvement of higher order LB area in neuronal stimulus discrimination.

There is a substantial literature on subdivisions of auditory cortical areas and their roles in processing complex sounds (Romani et al., 1982; Rauschecker et al., 1995; Eggermont, 1998; Bendor and Wang, 2008; Ghazanfar et al., 2008; Recanzone, 2008; Kuśmierek and Rauschecker, 2009; Bandyopadhyay et al., 2010; Kikuchi et al., 2010, 2014; Camalier et al., 2012; Sundberg et al., 2012; Niwa et al., 2013). In this study, we investigated one such complex sound, viz., a Coo, that can be represented spectro-temporally as containing higher harmonics of a specific fundamental frequency (Fig. 2), as opposed to a simple sound consisting of a single frequency. The animals were trained to respond to a stimulus that had no periodic temporal structure (pink noise), but that required them to allocate equivalent levels of attention to both simple and complex sounds (PTs and Coos, respectively). A traditional, trial-averaged analysis of the data indicated that the spike-onset latency for the PT was shorter in A1 than in LB (Kikuchi et al., 2014). However, there was a minimal difference in latency between A1 and LB for Coo sounds, a finding that may seem surprising from the perspective of serial hierarchical information processing. We argue that an effective way to tease out the entire processing architecture is to look at simultaneous measurements of inputs and output of a brain area using both spike and LFP recordings. We showed that stimulus-specific spike and LFP responses are present in A1 and LB, as found in previous studies (Ghazanfar et al., 2005, 2008). We then compared single-trial latencies from spike trains and LFPs at the same electrode and across different electrodes. This presents a unique way to extract the local functional connectivity in auditory cortex underlying complex sound processing.

Latency comparison has been used previously to estimate functional neural circuitry underlying complex tasks (DiCarlo and Maunsell, 2005; Hung et al., 2005; Buschman and Miller, 2007; Monosov et al., 2008). The key methodological innovation in the current paper is employing the AccLLR framework, which allows single-trial decoding of latencies from spike/LFP data (Fig. 5). Using AccLLR, we were able to evaluate latencies statistically within one session as well as compare them across sessions and thereby enhance the statistical power of our results. A somewhat similar approach based on the computation of a “surprise index” was proposed earlier by Hanes and colleagues (Hanes et al., 1995). For comparison, we also performed the latency analysis by applying the commonly used method employing a ms-by-ms rank sum test (Fig. 4). Comparison of Figures 4, 5 (AccLLR results) illustrate a dramatic improvement in statistical significance of results for the trial-by-trial analysis. The trial-by-trial analyses as well as pooled trial analysis (accuracy matched) confirm the pattern of results reported by Kikuchi et al. (2014): spike onset latencies were shorter in A1 than in LB for PTs but close to each other for Coos. Error rates from decoded LFPs were higher than corresponding spike-analysis sessions, although across sessions decoding was better than chance, indicating the robustness of the information contained in LFPs. Robust decoding using LFPs was also reported in earlier studies (Hung et al., 2005; Markowitz et al., 2011; Bansal et al., 2012).

### Functional neural circuitry underlying auditory processing

A central aim of the current study was to compare latencies of spike and LFP responses in two different contexts, at onset and during neuronal selection. Latencies were compared across stimuli (PT vs Coo) to investigate the stimulus-specific components. A key result from AccLLR analyses (both trial-by-trial and performance matched) was the nearly identical LFP onset latency in A1 for PTs and Coos and the very similar onset latencies in LB for these two stimulus categories (Figs. 4, 5). If we consider LFPs to be coupled more to inputs, the information related to the presence of an auditory stimulus in the environment arrives at both brain areas simultaneously. Previous studies demonstrated that A1 and LB receive inputs in parallel from subcortical structures, which may be the reason that there is little difference in LFP onset latencies across the two areas (Rauschecker et al., 1997; de la Mothe et al., 2006). In the case of sensory areas, where feed-forward connections dominate, relative spike latency can indicate a putative area’s contribution to information processing (VanRullen et al., 2005). In our findings, spike onset latency was usually longer than LFP onset latency in agreement with previous studies in sensory areas (Eggermont, 1998; Sundberg et al., 2012). We observed that the spike onset latency computed from trial-averaged data are shorter in A1 than in LB for PTs but not for Coos. The also followed this trend. Interestingly, spike onset latency for Coo in LB was shorter than spike onset latency for PT using both single trial and performance matched AccLLR analyses (although a clear trend was observed in the latter analysis that matched trial-by-trial results, the latency differences were not significantly different). This validates the view that the auditory cortex is organized into lower-order sensory areas (e.g., A1), relevant for coding simple features such as fundamental frequencies, and (relatively) higher-order LB areas for coding more complex auditory features (Rauschecker et al., 1995; Kikuchi et al., 2010). On the other hand, spike onset latencies for Coo in A1 and LB were not significantly different. This suggests that complex signals require more distributed resources for processing. The aforementioned findings were replicated when statistical analysis was applied to the data from each monkey individually (Fig. 5).

An important point to note here is that the single-trial latencies detected by AccLLR analysis are typically longer than trial-averaged latencies or ones obtained from pooling all trials and setting detection accuracy to 100%. In an earlier stimulus onset latency detection study, Banerjee et al. (2010) showed that latencies computed from trial-averaged AccLLRs can decrease by 15 ms at the expense of an increase in false-alarm rates. In our study, only the LFP onset latencies were very close among trial-averaged, pooled trial AccLLR and single trial AccLLR results. For spike onset latencies, the differences were maximal between trial-averaged and AccLLR measures and same pattern was followed in latency distributions from pooled trials. This indicates that LFPs may have the least variability in recording the presence of an auditory stimulus, and such tight time-locking is most likely due to the subcortical nature of the stimulus processing before it arrives in A1.

Area-specific properties in processing differences between stimuli can be investigated using selection latencies. Shorter LFP selection latencies in A1 compared to LB may reflect the hierarchical organization of these areas vis-à-vis stimulus-specific processing, e.g., dissociating simple (PT) from complex (Coo; Fig. 5). For both trial-averaged and trial-by-trial analysis, spike selection latency in LB was shorter than spike selection latencies in A1, indicating a stronger role of LB in processing stimulus-specific features. Combining this finding with the results from the onset latency analysis, we can dissociate the function of the two brain areas in computing different components of information processing in an environmental signal, i.e., just the presence of sound versus the detailed features of that sound. We did not observe a stimulus-specific difference, PT compared to Coo, in LFP selection latency in the two areas (*p* = 0.57 in A1, *p* = 0.01 in LB, latencies reported in Table 1). The effect was robust when the analysis was performed in individual monkeys as well as when latencies were computed by pooling all trials and applying the AccLLR framework (Fig. 5), although it was not present in the trial-averaged analysis from raw time series (Fig. 4). We thus conclude that at least some stimulus-specific information arrives serially in these two brain areas, contrary to what we observed for LFP onset latency. An alternative possibility is that the lower-order auditory area A1 receives feedback projections from LB or other higher-order areas. Spike selection latency in A1 was longer than the LFP selection latency when both trial-averaged and trial-by-trial analyses were performed on individual monkey data as well as on the population data. When detection threshold was set at 100% in pooled trials this difference in spike-LFP selection latencies in A1 was not observed.

On the other hand, spike selection latency in LB was comparable to the LFP selection latency, although there is a slight variability in this result when one examines the data on individual monkeys (Fig. 5). Monkey1 exhibited the general trend of spike selection latency being longer than LFP selection latency, just as in the case of onset latencies. However, Monkey2 showed slightly shorter spike selection latencies than LFP selection latencies in LB (Fig. 5). Earlier research has established that A1 and LB have strong reciprocal connections (de la Mothe et al., 2006; Hackett, 2011). Together, these data raise the possibility that LB has a top-down preparatory role for selection-related processing, whereas A1 is primarily involved in bottom-up gating of sensory signals.

### Future directions and limitations

Our study provides a design-analysis framework to support neurophysiological findings that could help address questions related to functional networks at both local area-specific scales and global interareal scales. Such studies would shed light on task-specific network mechanisms underlying complex behavior. One limitation of the current study is that it ignores the information about the endogenous neural states present in ongoing oscillations and how such processes affect extrinsic stimulus driven processing. A recent study has shown that neuronal areas separated across large distances whose activities are coherent may also exhibit lower latencies in information processing using AccLLR (Wong et al., 2016). The same framework could also be adapted to detect the timing of oscillatory-response onsets and phase differences from the electrical activity of nearby and distant populations. Finally, AccLLR can be applied to macroscopic neural recordings such as electroencephalogram (EEG), intracranial EEG, and magnetoencephalogram (MEG) to estimate network mechanisms and thereby inform a wider research community.

## Synthesis

Reviewing Editor: Satu Palva, University of Helsinki Decisions are customarily a result of the Reviewing Editor and the peer reviewers coming together and discussing their recommendations until a consensus is reached. When revisions are invited, a fact-based synthesis statement explaining their decision and outlining what is needed to prepare a revision will be listed below. The following reviewer(s) agreed to reveal their identity: Flavio Frohlich.

Synthesis

Both reviewers' found your study interesting but they also identified some major issues that should be clarified before publication. One of the major issues that was found problematic was that the eventual message of the study was unclear and the reviewers where uncertain of whether message of the paper is 1) validation of the method to extract connectivity, 2) improvements to an existing method to enhance statistical power, or 3) demonstration that naturalistic sounds activate auditory networks differently from pure tones/noise. The eventual message should be clarified and tested accordingly.

Additionally, it was thought that a more sensitive method, termed AccLLR, could be used for the trial average approach. This could resolve notable inconsistencies in important aspects of the findings, specifically, regarding the second monkey LFP results. Also another approach for averaging trials and measuring speed-accuracy trade off was suggested as well as of the inferences of the results which are based on the evoked potentials in the LFP and not the induced oscillations.

These major issues are specified in the reviewer comments below.

Major

Reviewer # 1

I am intrigued by the paper but I am not quite sure what the eventual message is.

(1) Validation of the method to extract connectivity? It seems to be the main aim, thus a validation is needed in the form of a detailed functional connectivity analysis of both the spiking and LFP data.

(2) Minor (?) improvements to an existing method to enhance statistical power? If so, this should be rewritten as a methods paper with synthetic datasets extensively tested and a clear quantification of the relative gain in statistical power.

(3) Demonstration that naturalistic sounds activate auditory networks differently from pure tones/noise? If so, many more control stimuli and new experiments are needed.

Reviewer # 2

This very interesting study examines the serial and hierarchical nature of auditory processing in the primary auditory cortex (A1) and lateral belt auditory cortex (LB). Macaque monkeys are trained to perform go/no-go discriminations when presented with pure tone and conspecific vocalizations - a Coo. A comprehensive analysis of the latencies of spiking and LFP activity to stimulus onset and stimulus discrimination is presented for each area. The pattern of results is reported across signal type (spike/LFP), stimulus type (simple/complex), information type (onset/discrimination) and area (A1/LB).

This work analyzes spike-LFP recordings to provide convincing evidence that A1 and LB operate to process sounds based on stimulus specific information that likely arises from the auditory thalamus, in a parallel fashion, and that discrimination of stimulus identity occurs earlier in LB than A1, consistent with hierarchical top-down-like computations. I am generally very enthusiastic about this study which is well reasoned, well-written and presents clearly significant findings. I have a few comments/suggestions for improvement

(1) The authors present results according to trial averages as well as on single trials using two different methods. The results are generally consistent, and indicate the single trial method is more sensitive. The use of two methods is a strength. This said, I would like the authors to report the results of the more sensitive method, termed AccLLR, using the trial average approach. I believe this will form a valuable contribution to the paper because it may resolve notable inconsistencies in important aspects of the findings. Specifically, the second monkey LFP results are somewhat slow for discrimination, and the discrimination time error bars are pretty broad indicating lack of power or inconsistent power in the test.

(2) At present, single trial responses yield correct performance that is better than chance, but importantly the performance is not 100%. If the authors detect events when averaging trials, they will achieve greater sensitivity. They can use this sensitivity to more precisely control the speed-accuracy trade-off inherent in latency analyses by measuring the first time when they can detect the signal with accuracy set to 100%. This will lead to a latency comparison between the two areas (A1/LB) and two signal types (spike/LFP) that more completely controls for the speed-accuracy trade-off. Any concern that remains reflects the choice of task (stimulus/difficulty) and recording strategy/bias (locations sampled) which can rightly form the subject of a different study.

(3) The authors carefully interpret the spike-LFP recordings but this is a complicated area. The authors use the idea that the LFP measures synaptic input which is attractive but not strictly true. The LFP also measures local synaptic processes that are not necessarily due to input. I would like the authors to make a clear statement that the inferences they make are likely to be valid because they are made based on the evoked potentials in the LFP and not the induced oscillations. Others have shown that evoked potentials in primary/early sensory areas, reflect current source-sink effects that arise from inputs while the same is not so clearly true for induced oscillations.

Minor comment

• I found the last sentence of the first discussion section paragraph to be hard to parse. I would rephrase.

## Author Response

Responses to Editor

‘Both reviewers’ found your study interesting but they also identified some major issues that should be clarified before publication. One of the major issues that was found problematic was that the eventual message of the study was unclear and the reviewers where uncertain of whether message of the paper is 1) validation of the method to extract connectivity, 2) improvements to an existing method to enhance statistical power, or 3) demonstration that naturalistic sounds activate auditory networks differently from pure tones/noise. The eventual message should be clarified and tested accordingly.

Thank you for handling our manuscript and giving us a chance to resubmit by clarifying our message. We are also grateful to the reviewers for pointing out issues related to the main message being unclear as well as the encouraging comments. While going through the points we recognize that the main problem in our first submission was that it was not clear what goals our study seeks to achieve. We have now clearly stated in Introduction as well as the Discussion that we are not proposing a new method but applying a technique to interpret the observations from an actual data set. Indeed our main focus is to understand the circuit mechanisms that exist within the substructures of the auditory cortex.

Additionally, it was thought that a more sensitive method, termed AccLLR, could be used for the trial average approach. This could resolve notable inconsistencies in important aspects of the findings, specifically, regarding the second monkey LFP results. Also another approach for averaging trials and measuring speed-accuracy trade off was suggested as well as of the inferences of the results which are based on the evoked potentials in the LFP and not the induced oscillations.

AccLLR and Trial-averaged measures serve different purposes and one is not necessarily a sensitive version of the other. Even though it is possible to detect the Onset latency for each stimulus category from trial averaged measures since the null distribution was the baseline condition, nonetheless, a stimulus-specific selection latency (selection latency for pure tone (PT) or Coo separately) cannot be defined for an ensemble of trials. While using trial averaged measures, there is one selection latency for PT and Coo discrimination computed over an ensemble of trials. Thus, the power of AccLLR is in extracting the latencies at the single trial level which leads to lower error bars accepting the fact that the nature of the accumulating evidence procedure makes the detection of latencies inherently slower and probablistic. As reviewer 2 suggested we have added the results from applying statistical thresholds on trial-averaged AccLLRs for consistency and completeness and also to compare the speed-accuracy aspect. These results are included in Fig 4b and Table 1. All inserts are marked in blue.

We acknowledge the issue about Selection latencies of a particular category not being defined at the level of trial averaged measures was not properly articulated in our Discussion as reviewer 2 pointed out. Now we added a statement of clarification in Discussion about this aspect.

We have also added a few edits in the text to make our message clearer (see below). In the main text we have marked all our edits in colored font.

2

_________________________________________________________________________________

Responses to Reviewer # 1

I am intrigued by the paper but I am not quite sure what the eventual message is. (1) Validation of the method to extract connectivity? It seems to be the main aim, thus a validation is needed in the form of a detailed functional connectivity analysis of both the spiking and LFP data.

Thank you for this comment. The purpose of this article is not to validate an already established method (Banerjee et al 2010, 2012; Wong et al 2017). The focus is on detecting if subdivisions of auditory cortex in macaque monkeys

1) receive information related to presence of sounds in environment in parallel or in hierarchically?

2) is the strategy changed during extraction of meaningful information about the content of the sound

using chronometry as suggested in the widely cited work of Di Carlo and Maunsell 2005. We have made the following changes in the Introduction to make these aspects clear.

lines 65-67: Additionally, the timing of input versus output of information processing in an area can be used to infer the role of this area in processing of a particular type of signal as well as the functional pathways involved in processing of the signal (DiCarlo and Maunsell, 2005).

Lines 86-88: However, do the two areas receive information about stimulus presence concurrently? Furthermore, are the finer features that allow discrimination of one signal from the other represented in the neural codes hierarchically?

(2) Minor (?) improvements to an existing method to enhance statistical power? If so, this should be rewritten as a methods paper with synthetic datasets extensively tested and a clear quantification of the relative gain in statistical power. We would like to clarify this is not a Methods paper. AccLLR as a method has been been published earlier by Banerjee, Dean Pesaran (Journal of Neurophysiology 2010, Frontiers in Computational Neuroscience 2012). More recently it was used in Wong, Fabiszak, Novikov, Daw, Pesaran (Nature Neuroscience 2016). We carefully delimit ourselves to the interpretations that can be made unequivocally from this method in the current revised manuscript.

(3) Demonstration that naturalistic sounds activate auditory networks differently from pure tones/noise? If so, many more control stimuli and new experiments are needed.

Indeed we have pure tones of 10 different frequencies, 10 fundamental matched Coo stimuli and pink noise stimuli that were cued as the Go Stimuli (see Kikuchi et al 2014). The trials become very much fewer in number and lack statistical power when sorted for each specific tone and corresponding tone matched-Coo. Nonetheless our question was also related to see if neural codes are found for category specific processing PT vs Coo. Here this variability of stimulus within a category (PT / Coo) helps us to overrule the possibility that the results in discrimination of PT from Coo are because of some specific representation of one Tone or one specific Coo stimulus.

3

Responses to Reviewer # 2 This very interesting study examines the serial and hierarchical nature of auditory processing in the primary auditory cortex (A1) and lateral belt auditory cortex (LB). Macaque monkeys are trained to perform go/no-go discriminations when presented with pure tone and conspecific vocalizations - a Coo. A comprehensive analysis of the latencies of spiking and LFP activity to stimulus onset and stimulus discrimination is presented for each area. The pattern of results is reported across signal type (spike/LFP), stimulus type (simple/complex), information type (onset/discrimination) and area (A1/LB). This work analyzes spike-LFP recordings to provide convincing evidence that A1 and LB operate to process sounds based on stimulus specific information that likely arises from the auditory thalamus, in a parallel fashion, and that discrimination of stimulus identity occurs earlier in LB than A1, consistent with hierarchical top-down-like computations. I am generally very enthusiastic about this study which is well reasoned, well-written and presents clearly significant findings. I have a few comments/suggestions for improvement

Thank you for the very encouraging comments. (1) The authors present results according to trial averages as well as on single trials using two different methods. The results are generally consistent, and indicate the single trial method is more sensitive. The use of two methods is a strength. This said, I would like the authors to report the results of the more sensitive method, termed AccLLR, using the trial average approach. I believe this will form a valuable contribution to the paper because it may resolve notable inconsistencies in important aspects of the findings. Specifically, the second monkey LFP results are somewhat slow for discrimination, and the discrimination time error bars are pretty broad indicating lack of power or inconsistent power in the test. We agree with the observation that a trial averaged measures from AccLLR should be reported. Hence we have included the results from obtaining the latencies using ms-by-ms t-tests on trial averaged AccLLRs. This is now included in fig 4b and Table 1. We have now performed ms-by-ms t-tests on raw time series also instead of rank sum as followed previously by Monosov et al study.

(2) At present, single trial responses yield correct performance that is better than chance, but importantly the performance is not 100%. If the authors detect events when averaging trials, they will achieve greater sensitivity. They can use this sensitivity to more precisely control the speed-accuracy trade-off inherent in latency analyses by measuring the first time when they can detect the signal with accuracy set to 100%. This will lead to a latency comparison between the two areas (A1/LB) and two signal types (spike/LFP) that more completely controls for the speed-accuracy trade-off. Any concern that remains reflects the choice of task (stimulus/difficulty) and recording strategy/bias (locations sampled) which can rightly form the subject of a different study.

The speed-accuracy trade-off aspects of AccLLR method were earlier reported in a study by Banerjee, Dean and Pesaran Journal of Neurophysiology 2010. This was done by creating bootstrapped estimates of raw data by taking a few trials and averaging them to generate a pseudo -trial. This approach targets a methodological question of how AccLLR performance varies when signal quality is changed. In context of the current empirical study this is somewhat ad-hoc because the pseudo-trial created by sub-sampling is not real data anymore and including this kind of analysis seems to draw

4

the focus away from the empirical findings.

(3) The authors carefully interpret the spike-LFP recordings but this is a complicated area. The authors use the idea that the LFP measures synaptic input which is attractive but not strictly true. The LFP also measures local synaptic processes that are not necessarily due to input. I would like the authors to make a clear statement that the inferences they make are likely to be valid because they are made based on the evoked potentials in the LFP and not the induced oscillations. Others have shown that evoked potentials in primary/early sensory areas, reflect current source-sink effects that arise from inputs while the same is not so clearly true for induced oscillations. We have rephrased the earlier mentioned in Introduction in this regard from

“While neuronal spike discharge is used as a measure of output processing in a putative brain area (Kruger and Becker, 1991, Middlebrooks et al., 1994, Nawrot et al., 2003, Buzsaki et al., 2012), local field potentials (LFPs) may carry information about the inputs coupled with local neuronal processing in a particular brain area (Gusnard et al., 2001, Nielsen et al., 2006, Buzsaki et al., 2012) and by extending this principle to multiple brain areas, aspects of the functional circuitry underlying behavior can be revealed (Hung et al., 2005, Banerjee et al., 2012) (Figure 1).”

to “While neuronal spike discharge is used as a measure of output processing in a putative brain area (Kruger and Becker, 1991, Middlebrooks et al., 1994, Nawrot et al., 2003, Buzsaki et al., 2012), local field potentials (LFPs) may carry information about the inputs coupled with local neuronal processing that need not be input-specific, in a particular brain area (Gusnard et al., 2001, Nielsen et al., 2006, Buzsaki et al., 2012) and by extending this principle to multiple brain areas, aspects of the functional circuitry underlying behavior can be revealed (Hung et al., 2005, Banerjee et al., 2012) (Figure 1).”

Minor comment • I found the last sentence of the first discussion section paragraph to be hard to parse. I would rephrase.

Rephrased. “Using two measures, onset latency for detecting the presence of sound in the environment and selection latency for identifying stimulus specific neural codes in primate auditory cortical areas we aim to characterize the functional pathways of underlying information processing.”

____________________________________________________________________________

Some additional changes for clarifying the main message:

1. We have replaced the definition Discrimination latency with Selection latency in the entire manuscript, because the animals are not behaviorally discriminating between PT an Coo stimulus. However, a neuronal selection for coding of one stimulus vs other takes place in their auditory cortex.

2. We have added a sentence in the Discussion as to why trial-average measures do not extract the stimulus -specific selection latencies- “The trial-averaged techniques do not allow the measure of stimulus-specific selection latencies since a distribution of ‘Pure tone trials’ is used to identify the time of selection from a distribution of ‘Coo trials’.”

5

3. We have added a statement about the limitation of our study in the last paragraph of the Discussion. “One limitation of the current study is that it ignores the information about the endogenous neural states present in ongoing oscillations and how such processes affect extrinsic stimulus driven processing.”
